# The Prognostic Role of PORT and EGFR Mutation Status in Completely Resected Stage IIIA/N2 Non-Small Cell Lung Cancer Patients with Postoperative Chemotherapy

**DOI:** 10.3389/pore.2021.1609898

**Published:** 2021-08-10

**Authors:** Hui Yang, Kunlun Wang, Shenglei Li, Yan Li, Ling Yuan

**Affiliations:** Department of Radiation Oncology, The Affiliated Cancer Hospital of Zhengzhou University, Zhengzhou, China

**Keywords:** EGFR, NSCLC, stage IIIA/N2, postoperative radiotherapy, prognostic factors

## Abstract

**Background:** The treatment choice for completely resected stage IIIA/N2 non-small cell lung cancer (NSCLC) patients is still controversial now. Our study aims to identify potential prognostic factors in stage IIIA/N2 NSCLC patients with complete surgical resection and postoperative chemotherapy.

**Methods:** In this study, we screened the stage IIIA/N2 NSCLC patients diagnosed in the Affiliated Cancer Hospital of Zhengzhou University from 2015 to 2019. Completely resected patients with postoperative chemotherapy (PCT) were enrolled. The univariate and multivariate COX proportional hazards regression analyses were used to identify the prognostic factors. The Kaplan-Meier survival curve was used to compare the disease-free survival (DFS) and overall survival (OS) in the subgroup analyses.

**Results:** 180 patients were collected, including 142 patients with PCT treatment alone and 38 patients with postoperative radiotherapy (PORT) treatment. The median DFS was 17.8 months (95% CI: 16.5–19.1 months) and the median OS was 50.6 months (47.4–53.9 months) in all the patients. The median DFS of the PORT group was significantly longer than the PCT group (38.7 vs 16.7 months, *p* < 0.001). Epidermal growth factor receptor (EGFR) mutation-positive patients had a worse DFS compared with EGFR mutation-negative patients (16.8 vs 18.0 months, *p* = 0.032). Possible prognostic factors were evaluated through univariate COX regression analysis. The further multivariate COX regression analysis showed that patients with PORT (HR: 0.318, 95% CI: 0.185–0.547, *p* < 0.001), EGFR mutation-negative (HR: 0.678, 95% CI: 0.492–0.990, *p* = 0.044), T1 (HR: 0.661, 95% CI: 0.472–0.925, *p* = 0.016), and lobectomy (HR: 0.423, 95% CI: 0.191–0.935, *p* = 0.034), had better DFS. The only independent prognostic factor of OS was the type of surgery (*p* = 0.013).

**Conclusion:** PORT might improve the DFS of stage IIIA/N2 NSCLC patients with complete surgical resection and PCT, but it cannot increase OS. Besides, EGFR mutation status, T stage, and type of surgery are possible independent prognostic factors for DFS, and type of surgery is associated with OS. These factors remain to be clarified in further studies.

## Introduction

Lung cancer is one of the most common cancer with the highest mortality in the world (1). Over 80% of patients are non-small cell lung cancer (NSCLC), and approximately 15% of patients were diagnosed as stage IIIA NSCLC (2). Stage IIIA NSCLC patients have heterogeneous clinical features, prognoses, and treatment choices.

For stage IIIA/N2 NSCLC patients, surgery is the most effective treatment. However, at least 30% of patients suffered local recurrence or distant metastasis within 5 years after complete surgical resection and the 5-years overall survival (OS) rates were only 15–20% (2-3). Thus, complete surgical resection combined with postoperative therapy is considered as the main treatment modality.

Postoperative chemotherapy (PCT) was usually conducted to overcome occult distant micrometastases and improve clinical outcomes (4). However, the role of postoperative radiotherapy (PORT) remains controversial now. Several clinical trials and retrospective analyses reported the benefit of adding PORT to PCT. PORT could improve the local control, 5-years survival rate, and OS time (5-6). Unfortunately, some other studies found that PORT failed to bring disease-free survival (DFS) or OS benefit (7-8). The randomized controlled trials (RCTs) showed that PORT could only increase local recurrence-free rate (9-10). Strong evidence supporting the use of PORT was needed.

Currently, the use of epidermal growth factor receptor tyrosine kinase inhibitor (EGFR-TKI) as adjuvant treatment in completely resected stage IIIA/N2 patients with EGFR mutation-positive is recommended by National Comprehensive Cancer Network (NCCN). EGFR-TKIs could provide better DFS than chemotherapy (11-12). However, whether EGFR mutation status is associated with survival time of completely resected IIIA/N2 NSCLC patients with PCT is unclear.

This study retrospectively analyzed the potential prognostic factors in stage IIIA/N2 patients treated with complete surgical resection and PCT, especially the role of PORT and EGFR mutation status.

## Methods

### Patient Selection

This study retrospectively collected patients diagnosed at the Affiliated Cancer Hospital of Zhengzhou University from January 2015 to December 2019. The inclusion criteria were: stage IIIA/N2 according to the eighth edition of the American Joint Committee on Cancer (AJCC) cancer staging system; completely resected; no neoadjuvant therapy; received PCT; no history of other malignant tumors. Patients were excluded when disease progression occurred before the completion of postoperative treatment; postoperative treatment was not completed; postoperative treatment involved targeted therapy or immunotherapy; had no enough follow-up data.

### Data Collection

A wide range of patient information was collected from the hospital database, including age, gender, pathological type, tumor size, positive lymph nodes, vascular tumor thrombus, EGFR mutation status, chemotherapy cycles and type of surgery.

### Statistical Analysis

SPSS Statistics software, version 26.0 (IBM Corporation, Armonk, NY, United States) was used for the analysis in this study. DFS was defined as the duration from operation to progression or end of the follow-up. OS was defined as the time from surgery to death or the last follow-up. The differences of baseline characteristics between patients of the PORT group and PCT group were compared by chi-square test. Corresponding two-tailed *p* values were evaluated and a *p*-value of <0.05 was considered statistically significant. Kaplan-Meier survival curve and log-rank test were used to compare the survival difference between every two groups. Univariate and multivariate analyses of prognostic factors were performed by Cox proportional hazards regression model. Factors with *p* < 0.1 in univariate analysis were selected into multivariate analysis to validate independent prognostic factors. The results of prognostic factors were expressed as hazard ratio (HR) with a 95% confidence interval (CI).

## Results

### Baseline Characteristics

180 patients were collected. The mean age of the total population was 57 years (range, 24–75 years), and 100 (55.6%) of the patients were male. The major histological type was adenocarcinoma (90.6%), and 70 (38.9%) patients were EGFR mutation-positive. 103 (57.2%) patients were stage T1, and 77 (42.8%) patients were stage T2. 85 (47.2%) patients had multistation N2 lymph nodes. Postoperative pathology showed that 115 (63.9%) patients had vascular tumor thrombus. 142 patients were treated with PCT, and 28 (15.6%) patients received chemotherapy for over four cycles. A small proportion of 3.9% of patients conducted pneumonectomy, other 173 (96.1%) patients performed lobectomy. The baseline characteristics of the patients are shown in Table 1.

**TABLE 1:** Baseline characteristics of patients.

Clinical feathers	Total (n = 180)	PORT (n = 38)	PCT (n = 142)	*p*
Gender				
Male	100 (55.6)	22	78	0.744
Female	80 (44.4)	16	64	
Age (year)	57 (24–75)	57 (38–73)	57 (24–75)	
<60	106 (58.9)	23	83	0.817
≥60	74 (41.1)	15	59	
Histological subtype				
Adenocarcinoma	163 (90.6)	35	128	0.956
Squamous carcinoma and others	17 (9.4)	3	14	
EGFR mutation				
EGFR mutation positive	70 (38.9)	14	56	0.724
Negative	110 (61.1)	24	86	
Pathological stage				
1	103 (57.2)	24	79	0.405
1a/1b/1c	3/39/61	3/7/14	0/32/47	
2	77 (42.8)	14	63	
2a/2b	53/24	11/3	42/21	
N2 lymph mode				
Multistation	85 (47.2)	17	68	0.730
Single station	95 (52.8)	21	74	
Vascular tumor thrombus				
Positive	115 (63.9)	26	89	0.513
Negative	65 (36.1)	12	53	
Chemotherapy cycle				
≤four cycles	152 (84.4)	37	115	0.083
>four cycles	28 (15.6)	1	27	
Type of surgery				
Lobectomy	173 (96.1)	37	136	1.000
Pneumonectomy	7 (3.9)	1	6	

### Prognostic Data

The median follow-up time was 31.5 months (range, 8.2–70.3 months), and 35 (18.6%) patients died at the last follow-up. The median DFS was 17.8 months (95% CI: 16.5–19.1 months) and the median OS was 50.6 months (47.4–53.9 months) in the enrolled patients (Table 2).

**TABLE 2:** Prognosis data in patients of different groups.

	Median DFS	95% CI	p	Median OS	95% CI	p
All	17.8	16.5–19.1	<0.001	50.6	47.4–53.9	0.370
PORT	38.7	27.3–50.2		52.7	48.4–57.0	
PCT	16.7	15.2–18.2		50.6	43.0–58.3	
EGFR mutation-positive	16.8	15.2–18.5	0.032	52.9	48.7–57.1	0.858
EGFR mutation-negative	18.0	12.5–23.5		50.0	42.2–56.8	

### Subgroup Analyses

Baseline characteristics had no statistical difference between the PORT and PCT groups (Table 1). The overall mortality was 19.5% in the PORT group, and 18.4% in the PCT group. The median DFS of the PORT group was significantly longer than the PCT group (38.7 vs 16.7 months, *p* < 0.001). The median OS in the PORT group was also longer, but had no statistically significant difference (52.7 vs 50.6 months, *p* = 0.370) (Table 2).

The DFS and OS were also compared according to the EGFR gene mutation status. We found that EGFR mutation-positive patients had a worse DFS (16.8 vs 18.0 months, *p* = 0.032) compared with EGFR mutation-negative patients. (Table 2). EGFR mutation-positive patients tended to have a better OS (52.9 vs 50.0 months), but the result was not statistically different.

### Univariate and Multivariate Analyses

In the univariate analysis of all patients, PORT (*p* = 0.010), EGFR mutation status (*p* = 0.032), T stage (*p* = 0.013), and type of surgery (*p* = 0.001) had prognostic value for DFS. Other factors, including gender, age, histological subtype, chemotherapy cycles, lymph node, and vascular tumor thrombus, were not associated with DFS. Type of surgery (*p* = 0.002) was the only significant factor for OS (Table 3).

**TABLE 3:** Univariate analysis of DFS and OS. Bold values were P values < 0.05.

	DFS			OS		
Factors	HR	95% CI	*p*	HR	95% CI	*p*
Gender (Male, Female)	1.005	0.721–1.400	0.976	1.105	0.664–1.838	0.701
Age (<60 years, ≥60years)	1.222	0.871–1.714	0.247	0.824	0.493–1.378	0.461
Histological subtype (Adenocarcinoma, Squamous carcinoma)	0.596	0.339–1.048	0.596	0.977	0.352–2.712	0.965
Chemotherapy cycle (≤four cycles, >four cycles)	0.839	0.543–1.297	0.430	0.934	0.483–1.805	0.839
N2 lymph mode (Single station, multstation)	0.815	0.961–1.339	0.815	0.868	0.518–1.456	0.592
Vascular tumor thrombus (Positive, Negative)	1.178	0.841–1.651	0.341	1.067	0.624–1.826	0.812
PORT (Yes, No)	0.520	0.353–0.765	**0.010**	0.695	0.312–1.546	0.372
EGFR mutation (Positive, Negative)	1.307	1.023–1.670	**0.032**	0.951	0.554–1.637	0.859
T stage (T1, T2)	0.657	0.471–0.915	**0.013**	0.637	0.376–1.078	0.093
Type of surgery (Lobectomy, Pneumonectomy)	0.270	0.125–0.584	**0.001**	0.264	0.112–0.623	**0.002**

In the further multivariate analysis, PORT (HR: 0.318, 95% CI: 0.185–0.547, *p* < 0.001), EGFR mutation status (HR: 1.475, 95% CI: 1.010–2.034, *p* = 0.044), T stage (HR: 0.661, 95% CI: 0.472–0.925, *p* = 0.016), and type of surgery (HR: 0.423, 95% CI: 0.191–0.935, *p* = 0.034), were independent prognostic factors for DFS, and type of surgery (HR: 0.329, 95% CI: 0.137–0.789, *p* = 0.013) was significant for OS (Table 4).

**TABLE 4:** Multivariate analysis of DFS and OS. Bold values were P values < 0.05.

	DFS			OS		
Factors	HR	95% CI	*p*	HR	95% CI	*p*
PORT (Yes, No)	0.318	0.185–0.547	**<0.001**			
EGFR mutation (Positive, Negative)	1.475	1.010–2.034	**0.044**			
T stage (T1, T2)	0.661	0.472–0.925	**0.016**			
Type of surgery (Lobectomy, Pneumonectomy)	0.423	0.191–0.935	**0.034**	0.329	0.137–0.789	**0.013**

## Discussion

Stage IIIA/N2 NSCLC patients have a high risk of local progression and distant metastasis after complete surgical resection. A multidisciplinary team with oncologists, surgeons, radiation oncologists, and radiologists is required to optimize the treatment options. The postoperative treatment is still controversial now.

A large-scale retrospective study analyzed 7,445 NSCLC patients from American Surveillance, Epidemiology, and End Results (SEER) database (5). 47% of patients were treated with PORT and had a higher 5-years OS rate than patients without radiotherapy (27.0 vs 20.0%), and mortality was decreased to 14.5% (*p* = 0.007). ANITA study reported that PORT increased the 5-years OS rate (from 34.0 to 47.0%) and median OS time (from 23.8 to 47.4 months) in stage IIIA/N2 NSCLC patients (6). The important role of PORT in stage IIIA/N2 NSCLC patients was also supported by plenty of RCTs and large population retrospective studies (13-18).

However, some clinical trials revealed that PORT could only improve the local control of stage IIIA/N2 NSCLC, but failed to bring DFS or OS benefit (7-8). The phase III RCT, NCT00880971, enrolled 364 completely resected stage IIIA/N2 NSCLC patients from 2009 to 2017 (9). Patients were randomly divided into PORT and PCT groups after four cycles of chemotherapy. This study founded that the PORT group had a better 3-years local recurrence-free rate (69.8 vs 62.4%, *p* = 0.03), but the median DFS (26.5 vs 22.7 months, *p* = 0.10), and the median OS (not reached vs 90.9 months, *p* = 0.94) in the two groups had no significant difference. The latest report of the Lung ART study (NCT00410683) enrolled 502 stage IIIA/N2 NSCLC patients in 11 years (10). It also showed that PORT reduced the local recurrence rate (from 46.1 to 25.0%), but could not improve DFS or OS.

Our study retrospectively analyzed the prognosis of stage IIIA/N2 NSCLC patients with complete surgical resection and PCT in our hospital within 5 years. We found that patients with PORT had better median DFS than patients with PCT alone (38.7 vs 16.7 months, *p* < 0.001). But the median OS was not significantly improved (52.7 vs 50.6 months, *p* = 0.370). We also identified that EGFR mutation status was an independent prognostic factor for DFS, and lobectomy was the only prognostic factor for OS.

We considered that PORT and PCT maybe not the most appropriate treatment for EGFR mutation-positive stage IIIA/N2 NSCLC patients. The ADJUVANT/CTONG1104 study (NCT01405079) revealed that gefitinib was better than chemotherapy in increasing the DFS (28.7 vs 18.0 months, HR = 0.60, 95% CI: 0.42–0.87, *p* = 0.0054) in completely resected stage II-IIIA (N1-N2) EGFR mutation-positive NSCLC (11). The ADAURA study (NCT02511106) also reported the advantage of osimertinib in DFS of completely resected early-stage (stage II to IIIA) NSCLC patients with EGFR mutation-positive (not reached vs 19.6 months; HR = 0.17, 99.06% CI: 0.11–0.26, *p* < 0.001) (12). Based on these studies, postoperative tyrosine kinase inhibitors (TKIs) seemed to be more useful for EGFR mutation-positive stage IIIA/N2 patients.

Our study has several limitations. First, it was a retrospective, single-center study with a small sample size. The selection of patients might affect the analysis results and bring bias. Second, the prognostic value of radiotherapy sequence, radiotherapy dose, radiotherapy target, and the duration from surgery to radiotherapy were not fully analyzed in this study which will reduce the effectiveness. We look forward to more multi-center RCT to provide high-quality evidence for the prognostic factors and treatment decisions of stage IIIA/N2 NSCLC patients.

In conclusion, PORT may increase the DFS, not OS in IIIA/N2 NSCLC patients. EGFR mutation status and T stage were also possible prognostic factors for DFS. And patients with lobectomy may have longer DFS and OS compared to pneumonectomy. However, large-scale studies are needed to further clarify the prognostic factors.

## Data Availability

The original contributions presented in the study are included in the article/supplementary material, further inquiries can be directed to the corresponding author.
